# Sensitive Detection of Heregulin-α from Biological Samples Using a Disposable Stochastic Sensor Based on Plasma Deposition of GNPs–AgPs’ Nanofilms on Silk

**DOI:** 10.3390/life11090894

**Published:** 2021-08-29

**Authors:** Sorin Sebastian Gheorghe, Catalina Cioates Negut, Marius Badulescu, Raluca Ioana Stefan-van Staden

**Affiliations:** 1 Faculty of Applied Chemistry and Material Science, Politehnica University of Bucharest, 060021 Bucharest, Romania; sebastiangheorghe09@yahoo.com; 2 Laboratory of Electrochemistry and PATLAB, National Institute of Research for Electrochemistry and Condensed Matter, 202 Splaiul Independentei St., 060021 Bucharest, Romania; 3 Low Temperature Plasma Laboratory, National Institute for Lasers, Plasma and Radiation Physics (NILPRP), 409 Atomistilor St., 077125 Magurele, Romania; marius.badulescu@inflpr.ro

**Keywords:** disposable stochastic sensors, brain tumor, silk, graphene nanoplatelet–silver particles composite, heregulin-α

## Abstract

A composite material comprised of graphene nanoplatelet and silver particles (GNPs–AgPs) was used for the deposition of GNPs–AgPs’ nanofilms with cold plasma on silk. α-Cyclodextrin was used as a modifier of the active surface of the disposable sensor. The disposable stochastic sensor was used in screening tests for the assay of heregulin-α in whole blood and tissue samples. The disposable stochastic sensor showed a low limit of determination (4.10 fg mL^−1^) and can be used with high sensitivity on a wide concentration range (4.10 fg mL^−1^–0.04 µg mL^−1^). The screening method was validated against ELISA when good correlations (confirmed also by the *t*-test) were obtained.

## 1. Introduction

Heregulins (HRGs) are members of the epidermal growth factor (EGF) family of peptides. Based on differences in the C-terminal portion of EGF, such as in terms of domain, HRGs are usually classified into two groups: HRG-α and HRG-β [1]. The HRG-α plays an important role in the development, regeneration, and tumor formation of the nervous system, including stimulation of acetylcholine receptor synthesis [2] and mitogenesis for Schwann cells, and therefore can provide good information regarding brain tumors [3]. This has been shown to be difficult to treat due to the biological characteristics in the types of cancers, which frequently limit its evolution. The developing, genetic, epigenetic, and microenvironmental characteristics of the brain make these cancers resistant to new and conventional treatments [4,5]. In recent years, there has been a slight increase in the survival of patients diagnosed with brain tumors. The main therapeutic methods are surgery, chemotherapy, and radiotherapy [6]. In the biomedical fields, the detection of biomarkers is the most researched aspect; these are used as indicators of biological and pathological processes in the body, and the concentration levels can provide information about a specific disease. Biomarkers can be detected in biological samples such as blood, urine, or various tissues. It is important to have very sensitive methods of analysis available to diagnose diseases at an early stage. Such a method requires simple and small devices for sensitive and reliable measurements of biomarkers, with reduced costs and simplification of the diagnosis by eliminating the need for medical staff [7]. In addition, HRG-α would regulate growth, invasion, and angiogenesis through either the overexpression or activation of an autocrine or paracrine loop, and would also act as a ligand for the HER-3 and HER-4 receptors. Due to the importance of HRG-α in the diagnosis and follow-up of treatments for brain cancer, there is a huge need to develop fast screening methods and tools for its detection and quantification in biological samples, such as whole blood and brain tumor tissue samples.

Stochastic sensors are very good alternatives to classical electrochemical sensors, as they are able to make reliable qualitative and quantitative analyses [8]. To date, they were successfully used for biomedical analysis [9,10,11,12]. The principle of stochastic sensors is based on current conductivity. When a potential is applied, the biomarker moves inside the channel, blocking it, and the current drops to zero until the molecule of the biomarker is inside the channel (the time needed for the biomarker to move inside the channel is called the signature of the biomarker t_off_ and depends only on the parameters of the biomarker, such as the length, unfolding capacity, and velocity of moving inside the channel). Inside the channel redox, processes take place and the polarity of the biomarker changes (the time needed for these processes is called t_on_ and is used as a quantitative parameter for the determination of the response characteristics of the stochastic sensor and also, based on its value, for the determination of the concentration of the biomarker in the biological sample). After this step, the biomarker (with the polarity changed) moves out of the channel, producing a new t_off_. Therefore, a disposable stochastic sensor was proposed in this paper for the detection and quantification of HGR-α in biological samples, such as whole blood and brain tumor tissue.

The novelty of this paper was the design of the disposable stochastic sensor: a composite material comprised of graphene nanoplatelet and silver particles (GNPs–AgPs) was used for the deposition of GNPs–AgPs’ nanofilms with cold plasma on silk. The resulting modified silk pieces were coupled with pieces covered with Ag/AgCl nanofilms (as reference electrodes), as well as with pieces covered with Pt nanofilms (as auxiliary electrodes). α-Cyclodextrin was further used for modification of the GNPs–AgPs’ nanofilms because this has the pores needed to obtain a signal specific for the stochastic sensors, as previously seen [13,14]. The resulting combined disposable stochastic sensor was used for screening tests of whole blood and brain tumor tissue samples.

## 2. Materials and Methods

### 2.1. Materials and Reagents

**Figure 1:**
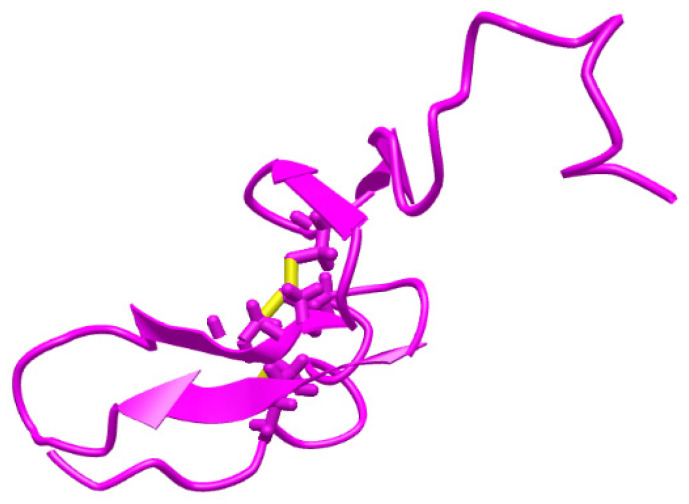
Structure of heregulin-α [15].

Heregulin-α (HGR-α) (Figure 1 [15]), α-cyclodextrin (α-CD), sodium phosphate dibasic heptahydrate, and sodium phosphate monobasic monohydrate were purchased from Sigma-Aldrich (Milwaukee, Wisconsin, USA). The reagents were of analytical grade and the solutions were prepared with deionized water obtained from a Direct-Q3 Water Purification system (Millipore Corporation, Bas-Rhin, France). HRG-α solutions of different concentrations were prepared in buffer solution (PBS 0.1 mol L^−1^, pH = 7.4) using the serial dilution method. The HRG-α solutions were used for 1 month; when they were not used for measurements, the solutions were stored in a fridge (2–8 °C). Graphene nanoplatelets (GNPs) was purchased from Nanografi (number: 7782-42-5) and used without further treatments. Silver particles (AgPs) of 99.9 % with different dimensions and size ranges (50–100 µm) were obtained in-house by mechanical grinding of bulk metal and were used without further purification. Throughout the procedures, double deionized water was used to obtain the composite paste solution of both materials.

### 2.2. Apparatus and Methods

For the stochastic measurements, an AUTOLAB/PGSTAT 12 (Metrohm, Utrecht, the Netherlands), linked to a computer with GPES software, was used. The combined stochastic disposable sensor was used for all measurements, performed at room temperature inside the Faraday cage. The chronoamperometric method was used at a fixed potential (125 mV vs. Ag/AgCl). The pH measurements were performed using a Mettler Toledo (Greifensee, Switzerland) pH/mv meter, model Seven Compact.

### 2.3. Design of the Disposable Stochastic Sensors

**Figure 2:**
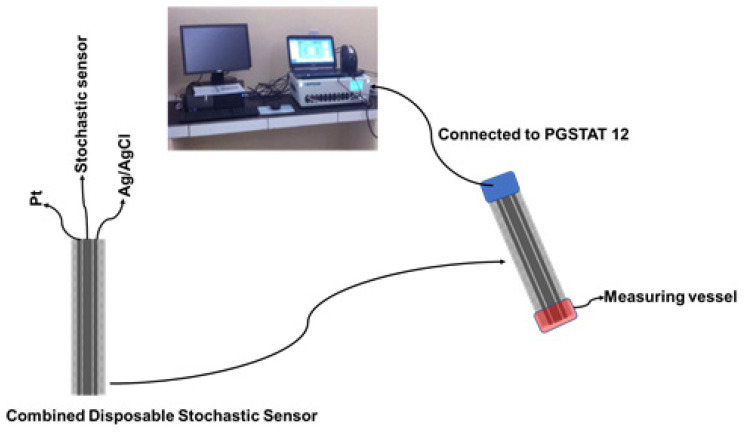
The design of the combined disposable stochastic sensor and its utilization for measurements.

The GNPs-AgNPs composite materials were obtained by mixing graphene nanoplates together with silver nanoparticles in a mass ratio of 2:1 with double deionized water (10 mL) to form a composite material. After that, the desired composite material was dried and heated at 100 °C in a vacuum chamber until the paste was transformed into a solid block. The coated composite nanofilms were synthesized using a cold plasma system by the thermionic vacuum arc method [16,17,18] onto organic-based substrates, such as silk textiles, with the size of 210 mm × 297 mm. At the outset, we implemented an external cleaning protocol for the organic-based substrates in order to remove contaminants such as particles. The deposition was carried out for 20 min under vacuum (1.3 × 10^−5^ mBar) with the substrate rotation at spin speed (50 rpm) in order to form a thin film with a high uniform thickness. The electrical parameters of the plasma were: 53 A filament current, 1.8 A plasma current, and 200 V plasma voltage. A 1.00 × 10^−3^ mol L^−1^ solution of α-cyclodextrin was added on top of the silk pieces covered with the GNPs-AgNPs’ nanofilms; after the silk pieces were immersed for two hours, they were dried for 24 h. When not in use, the disposable stochastic sensors were kept at room temperature in a dry place. Each disposable sensor was cable of being used for a maximum of 10 measurements. The combined disposable sensor containing also the printed Ag/AgCl (as a reference electrode) and Pt (as an auxiliary electrode) used the same technology as shown in Figure 2.

### 2.4. Stochastic Mode

All measurements were performed using the stochastic mode at 25 °C. A potential of 125 mV vs. Ag/AgCl was applied. Diagrams containing t_off_ and t_on_ parameters were recorded (Figure 7). After the identification of HGR-α, by identification of its t_off_ value, the value of t_on_ was read in between two consecutive t_off_ values. The t_on_ value was connected with the concentration of HRG-α through the equation: 1/t_on_ = a + b × C_HRG-α_. The equation of calibration was determined using the linear regression method. For the biological samples, the t_on_ values were read in the diagrams and used the equations of calibration, and the unknown concentrations of HRG-α were determined.

### 2.5. Samples

Seventeen biological samples were collected from confirmed patients with brain cancer in accordance with the procedures specified in the Ethics Committee, with the approval number 65573/14.12.2018 as awarded by the University Emergency Hospital from Bucharest. For these, written consent was obtained from all patients. All tissues were frozen instantly after resection and stored at temperatures of −80 °C. Over the wet brain tumor tissue samples, 1.0–2.0 mL of PBS pH = 7.4 was added. The whole blood samples (1.0–2.0 mL) were used for screening tests using the proposed disposable stochastic sensor as collected from patients without any pretreatment.

## 3. Results and Discussion

### 3.1. Characterization of the Material Used for the Design of the Disposable Stochastic Sensor

**Figure 3:**
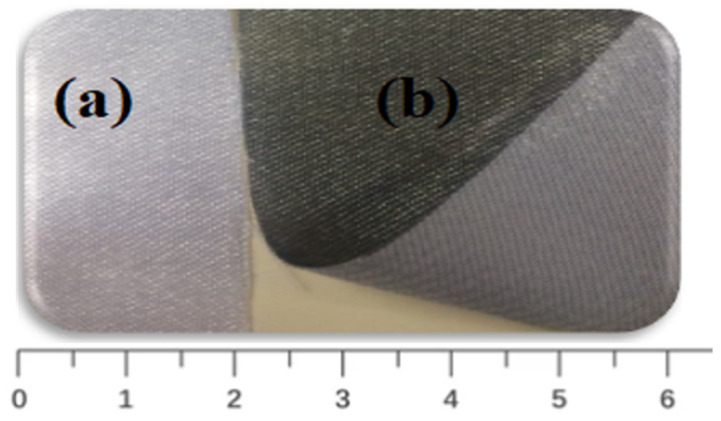
Photographs of (**a**) uncoated and (**b**) GNPs–AgNPs’ composite nanofilms coated onto silk textile substrates.

Digital photos of coated and uncoated GNPs–AgNPs’ nanocomposite films on one side of the planar surface of a silk textile substrate are shown in Figure 3.

**Figure 4:**
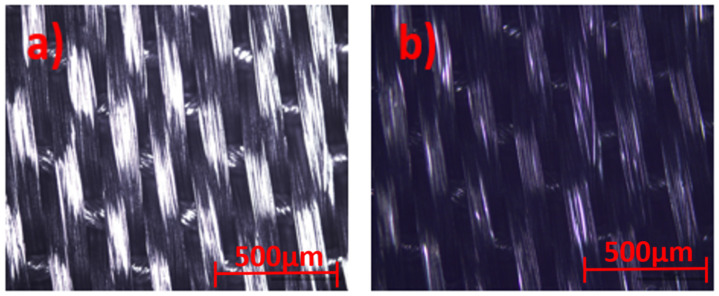
Optical microscope view of (**a**) uncoated and (**b**) GNPs–AgNPs’ nanocomposite films coated onto silk textile substrates.

Optical microscope images for the uncoated and coated silk textile surfaces are shown in Figure 4.

**Figure 5:**
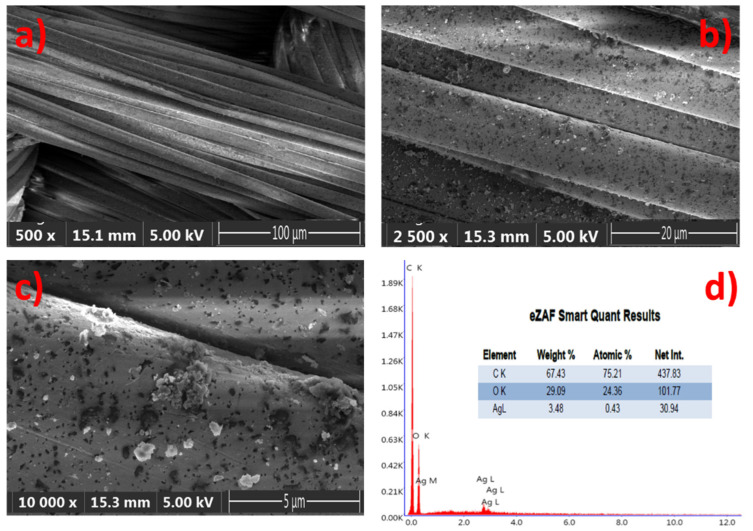
Composite nanofilms deposited onto silk textile substrates: (**a**–**c**) top view scanning an electron microscope (SEM) image of the same sample with different magnification and (**d**) both the EDS spectrum and elemental composition of the sample.

The samples were illuminated by reflected techniques and the images are directly viewed at 5× magnification, with a resolution of 500 µm, in order to provide a good textural shape. Scanning Electron Microscopy (SEM) images of the GNPs–AgNPs’ nanocomposite films on silk textile substrates are shown in Figure 5. The presence on the C layer and Ag-C composite layer of a large number of nanoparticles distributed over the surface of the coated substrate can be depicted with different magnification, as shown in Figure 5a–c.

The displayed images reveal the coated thin films’ uniformity on the rough surfaces with a high density of well-dispersed GNPs–AgNPs’ composite nanofilms. Moreover, the elemental analysis of the composite nanofilms was performed by using energy-dispersive-X-ray spectroscopy (EDS) and the spectra are presented in Figure 5d. Figure 5d reveals a typical EDS atomic mapping of C, O, and silver on the surfaces of the silk textile substrates coated with the Ag-C composite layer. The distribution of the various elements can be observed in addition to the C and O contents, which mainly attribute from the silk textile and no other impurities were detected. The EDS spectrum results confirm that Ag, C, and Ag-C are successfully deposited on the surface of the silk textile substrates in consistency with the proposed composition.

### 3.2. Response Characteristics of the Disposable Stochastic Sensors

**Figure 6:**
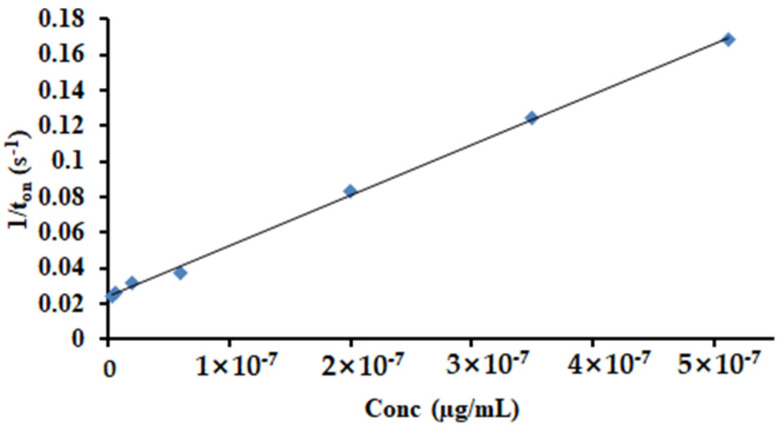
The calibration curve for the disposable stochastic sensor.

The response characteristic of the disposable stochastic sensor concerns that the signature of HGR-α was 1.4 s and the linear concentration range was between 4.10 fg mL^−1^ and 0.04 µg mL^−1^, with a sensitivity of 7.21 × 10^5^ s µg mL^−1^ and a limit of quantification of 4.10 fg mL^−1^. The equation of calibration was 1/t_on_ = 0.04 + 7.21 × 10^5^ × C, (<C> = µg mL^−1^ and <t_on_> = s) (Figure 6) and the correlation coefficient r = 0.9998.

**Figure 7:**
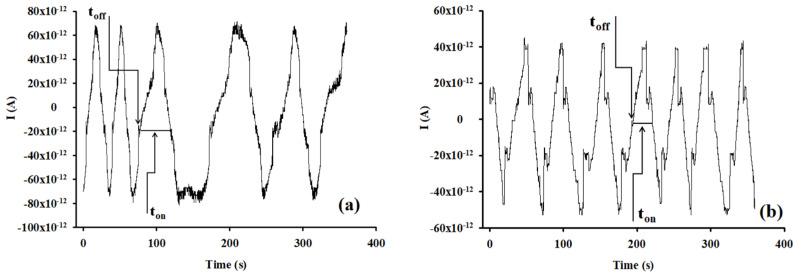
Types of diagrams recorded for the detection and quantitative determination of HRG-α in (**a**) whole blood and (**b**) tumoral brain tissue obtained using the screening method based on the disposable stochastic sensor.

The shape of the diagrams shown in Figure 7 (examples of diagrams obtained for the screening of biological samples) represent that the binding process is reversible: alternative up and down peaks are obtained and also there is no biofouling.

### 3.3. Selectivity of the Disposable Stochastic Sensor

The selectivity of the proposed disposable stochastic sensor was checked against neurotransmitters such as dopamine, epinephrine, and glutamate. The signatures (t_off_ values recorded) of the analytes served for the determination of selectivity; the signatures obtained for dopamine (2.3 s), epinephrine (1.0 s), and glutamate (0.3 s) are different than the one recorded for HGR-α (1.4 s), proving that the proposed disposable stochastic sensors were selective versus these neurotransmitters.

### 3.4. Screening Method for the Detection and Quantification of Heregulin-α in Whole Blood and Tissue Samples

The validation of the disposable stochastic sensor was done against ELISA, which is the method used in clinical laboratories for the assay of HGR-α using biological samples (whole blood and brain tumor tissues). The results of the determination of HGR-α in whole blood (Table 1) and brain tumor tissue samples (Table 2) obtained using the screening method based on the disposable stochastic sensor, as well as through using ELISA, showed a very good correlation between the two methods of analysis.

A paired *t*-test method was conducted for further validation of the screening method used for the detection and quantification of HGR-α. The paired *t*-tests were performed at a 99.00% confidence level. All calculated values for the paired *t*-test at the 99.00% confidence level were less than the tabulated theoretical value of 4.032 (Table 1 and Table 2). Accordingly, there was no statistically significant difference between the results obtained using the proposed screening method and ELISA, at a 99.00% confidence level, for the assay of HRG-α in whole blood and tumoral brain tissue samples. Accordingly, the screening method can be validated for the assay of HRG-α in whole blood and tumoral brain tissue samples.

## 4. Conclusions

A disposable stochastic sensor based on a nanofilm of a composite material comprised of graphene nanoplatelets and silver particles deposited on silk, and modified with α-cyclodextrin was designed, characterized, and validated for the assay of HRG-α in biological samples. The proposed sensor exhibited high sensitivity and selectivity on a wide concentration range. The limit of quantification for the disposable stochastic sensor, with an fg mL^−1^ magnitude order, is similar to that for 3D stochastic microsensors based on carbon nanotubes [19]. The sensor exhibited feature utilization in clinical laboratories and theaters for the fast detection and quantification of HRG-α in order to have a fast and early diagnosis of brain cancer and cancer metastasis. Compared with ELISA (the standard method used to date for the assay of HRG-α), the proposed method is faster, more reliable, cost-effective, and easy to use even in theaters when fast diagnosis is needed.

## Figures and Tables

**Table 1:** Determination of HRG-α in whole blood samples using the disposable stochastic sensor and ELISA.

Sample Number	pg mL^−1^, HRG-α	ELISA
	Disposable Stochastic Sensors	
1	7.17 ± 0.11	7.35
2	2.90 ± 0.25	2.19
3	7.42 ± 0.10	7.50
4	2.59 ± 0.13	2.20
5	6.48 ± 0.13	6.53
6	5.21 ± 0.12	4.73
7	560.30 ± 0.10	553.23
8	6.02 ± 0.15	5.98
9	4.47 ± 0.12	4.21
10	1.37 ± 0.10	1.15
11	19.74 ± 0.13	19.50
12	131.07 ± 0.14	129.15
*t*-test	2.19	-

**Table 2:** Determination of HRG-α in brain tumor tissue samples using the disposable stochastic sensor and ELISA.

Sample Number	pg mL^−1^, HRG-α	ELISA
	Disposable Stochastic Sensors	
1	153.60 ± 0.18	160.03
2	486.56 ± 0.13	430.15
3	690.50 ± 0.13	690.12
4	999.92 ± 0.15	993.15
5	18.66 ± 0.11	18.50
*t*-test	2.08	-
